# Chemoradiotherapy Versus Chemotherapy Alone for Advanced Esophageal Squamous Cell Carcinoma: The Role of Definitive Radiotherapy for Primary Tumor in the Metastatic Setting

**DOI:** 10.3389/fonc.2022.824206

**Published:** 2022-03-30

**Authors:** Li-Qing Li, Qing-Guo Fu, Wei-Dong Zhao, Yu-Dan Wang, Wan-Wan Meng, Ting-Shi Su

**Affiliations:** Department of Radiation Oncology, Guangxi Medical University Cancer Hospital, Nanning, China

**Keywords:** esophageal squamous cell carcinoma, advanced, metastatic, chemoradiotherapy, definitive radiotherapy, survival

## Abstract

**Introduction:**

The role of definitive radiotherapy in advanced esophageal squamous cell carcinoma (ESCC), especially in the metastatic setting, remains unclear. Therefore, we aimed to investigate the efficacy of chemoradiotherapy (CRT) versus chemotherapy (CT) alone in these selected patients.

**Methods:**

We retrospectively evaluated 194 newly diagnosed advanced ESCC who underwent definitive CRT or CT alone, including 97 patients with locally advanced and 97 patients with distant metastatic disease. Cumulative overall survival (OS) and progression-free survival (PFS) were evaluated with a log-rank test. Propensity score matching was used to simulate random allocation. In addition, we performed subgroup analysis in the locally advanced and metastatic disease.

**Results:**

After matching, 63 well-paired patients were selected. The adjusted median OS (12.5 vs. 7.6 months, *p* = 0.002) and PFS (9.0 vs. 4.8 months, *p* = 0.0025) in the CRT group were superior to that in the CT-alone group. Further subgroup analysis revealed that CRT conferred survival benefits to both locally advanced and metastatic cohorts. For patients with distant metastasis, median OS (12.9 vs. 9.3 months, *p* = 0.029) and PFS (9.9 vs. 4.0 months, *p* =0.0032) in the CRT group were superior to that in the CT-alone group. In a multivariate Cox regression analysis of the entire cohort, additional definitive radiotherapy was independently associated with better OS (*p* = 0.041) and PFS (*p* = 0.007).

**Conclusions:**

In both locally advanced and metastatic ESCC, additional definitive-dose radiotherapy was associated with improved clinical outcomes. Therefore, more consideration should be given to its application in the metastatic setting.

## Introduction

Esophageal cancer (EC) is the seventh most common cancer worldwide and the sixth leading cause of cancer-related death, with approximately 572,000 patients diagnosed in 2018 (1). The prognosis of metastatic EC is inferior, with a 5-year survival rate lower than 5% (2). Definitive radiotherapy (RT) with a dose greater than or equal to 50.4 Gy to the primary tumor is the mainstay of treatment and provides effective symptomatic relief for locally advanced EC (3–5). Since the RTOG 85-01 study, radical chemoradiotherapy (CRT) with a radiation dose of 50 Gy has been established as a curative treatment paradigm for locally advanced patients without evidence of distant metastasis (6). Subsequent clinical trials have further confirmed the clinical efficacy of this combination regimen (7, 8), which is now the standard first-line regimen for patients with locally advanced EC (9, 10). However, the current guidelines generally do not recommend aggressive radiotherapy for the primary tumor in patients with metastatic EC. The latest Chinese Society of Clinical Oncology guideline recommended only system therapy for metastatic EC (11). In the Pan-Asian adapted ESMO Clinical Practice Guidelines, RT was recommended only for palliative care to relieve patients’ dysphagia with metastatic EC (12). Systemic chemotherapy remains the cornerstone treatment for metastatic EC patients, with a median survival time of only 8–12 months (13–15). However, whether combined chemotherapy and aggressive radiotherapy can improve the survival of metastatic esophageal squamous cell carcinoma (ESCC) remains unclear. Therefore, in the present study, we aimed to investigate the efficacy and safety of chemotherapy-based definitive radiotherapy (≥50.4 Gy) in prolonging the survival of patients with advanced ESCC, particularly those with organ metastases.

## Materials and Methods

### Study Design and Patients

We retrospectively reviewed patients with newly diagnosed advanced ESCC who received CRT or CT alone at the Guangxi Medical University Cancer Hospital between June 2010 and May 2020. The institutional ethics committee approved this study, and informed consent was waived by the board. The eligibility criteria were as follows: (1) ESCC confirmed by histology; (2) clinically confirmed advanced disease (stage IVa or IVb) according to the 8th edition AJCC staging system (16); (3) Eastern Cooperative Oncology Group (ECOG) score 0–2; (4) no history of previous thoracic radiotherapy; (5) received definitive-dose (≥50.4Gy) radiotherapy for primary tumor for the CRT cohort; and (6) received no concurrent targeted therapy or immunotherapy.

### Chemotherapy and Radiotherapy Treatment

For all patients, two- or three-drug cisplatin-based chemotherapy was administrated at 3-week intervals for up to 6 cycles as first-line therapy. For patients undergoing CRT, definitive-dose radiotherapy was administrated synchronously with 2 to 3 cycles of cisplatin-based chemotherapy. Radiotherapy was performed with intensity-modulated radiotherapy using a 6-MV linear accelerator (Elekta Synergy, Stockholm, Sweden) at five fractions per week. The gross tumor volume (GTV) and metastatic lymph nodes (GTVnd) were delineated with visible lesions based on contrast-enhanced simulation CT, PET, and endoscopic evaluation results. The clinical target volume (CTV) was defined as a 0.5-cm horizontal expansion from GTV/GTVnd, a 3–5-cm craniocaudal margin from GTV, and a 0.5-cm craniocaudal margin from GTVnd. The planning target volume (PTV) was determined by adding a 0.5-cm margin to the CTV. A median total dose of 60 Gy (range, 56–66 Gy) with a median per dose of 2.0 Gy (range, 1.8–2.2 Gy) in a median fraction of 30 (range, 25–33) was prescribed to the PGTV for five consecutive days in a given week. The dose constraints for organs at risk (OARs) were as follows: (1) lung: the whole lung V20 <28%, V30 <20%, and Dmean <15–17 Gy; (2) spinal cord: Dmax <45 Gy; and (3) heart: V40 <30% and Dmean <30 Gy.

### Follow-Up and Statistical Analysis

For posttreatment follow-up, enhanced CT and upper gastrointestinal endoscopy were reevaluated 1 month after treatment and every 3 months after that. Progression-free survival (PFS) was defined as the period between the date of initial treatment until disease progression or recurrence or death. Overall survival (OS) was defined as the period from initial therapy to censor or death. OS and PFS rates were evaluated using the Kaplan–Meier method with the log-rank test. Continuous variables were compared with the Student’s t-test, while categorical variables were compared with Fisher’s exact or Pearson’s χ^2^ test. We performed multivariate Cox regression analysis to identify clinical variables independently associated with PFS and OS, and factors with p < 0.05 in the univariate Cox regression analysis were included. Statistical analysis was undertaken using R version 4.0.2 software, and p-values <0.05 were considered statistically significant.

To minimize potential selection bias and confounders, propensity score matching (PSM) was used to control for differences in baseline characteristics. Using a caliper of width equal to 0.2 without replacement, patients in the entire cohort were matched at a 1:1 ratio to simulate random allocation. Covariates entered into the propensity model included body mass index, ECOG score, TNM stage, number of metastatic sites, absolute neutrophil count, and albumin level. All baseline covariates were balanced in the locally advanced disease and metastatic disease subgroups. Therefore, PSM was not performed in the subgroup analysis.

## Results

### Patient Characteristics

A total of 194 patients with advanced ESCC were deemed eligible and assessed. Among them, 97 patients (50%) were locally advanced, and 97 patients (50%) had distant metastasis. The majority of patients with distant metastasis had a low systemic tumor burden. Seventy-seven (79.4%) patients had only one metastatic site (40.2%, 16.9%, 14.3%, 14.3%, and 14.3% of these patients presented with metastasis in the non-regional lymph node, lung, liver, bone, and others, respectively), and 14 (14.4%) patients had two metastatic sites. Merely 6 (6.2%) patients had at least three or more metastatic sites. A total of 101 patients (52.1%) received CRT, and 93 patients (47.9%) received CT alone. The median cycles of chemotherapy for the entire cohort were 3 (1–6 cycles). Before propensity score matching, patients in the CT-alone group had significantly worse baseline characteristics compared to those in the CRT group, with a lower body mass index (mean 20.3 vs. 21.5 kg/m^2^, *p* = 0.01), poorer physical performance (ECOG score 2: 8.6% vs. 0%, *p* = 0.011), greater tumor burden (stage IVb: 65.6% vs. 35.6%, *p* = 0.000, and distant metastatic sites ≥3: 6.4% vs. 0%, *p* = 0.001), lower absolute neutrophil count (mean 5.9 vs. 5.1 × 10^9^/l, *p* = 0.022), and lower albumin level (mean 35.5 vs. 36.7 g/l, *p* = 0.047). After matching, 63 well-paired patients were selected. There were no significant differences between the CRT group and the CT-alone group in baseline characteristics after PSM, as shown in **Table 1**.

**Table 1:** Patient characteristics before and after PSM in the CRT and CT alone groups.

		Before PSM			After PSM		
Factor	Level	CT (n = 93)	CRT (n = 101)	p	CT (n = 63)	CRT (n = 63)	p
**Gender**	Male	86	91	0.741	58	57	1.000
	Female	7	10		5	6	
**Age (yrs, mean ± SD)**		57.4 ( ± 0.9)	56.1 ( ± 0.9)	0.298	56.3 ( ± 1.0)	55.0 ( ± 1.1)	0.149
**BMI (m^2^/kg, mean ± SD)**		20.3 ( ± 0.3)	21.5 ( ± 0.3)	0.010	20.8 ( ± 0.4)	21.0 ( ± 0.3)	0.687
**ECOG**	0	8	10	0.011	5	3	0.715
	1	77	91		58	60	
	2	8	0				
**Smoking**	No	25	38	0.149	17	23	0.339
	Yes	68	63		46	40	
**Drinking**	No	25	32	0.565	17	22	0.441
	Yes	68	69		46	41	
**Family history**	No	89	92	0.320	61	59	0.676
	Yes	4	9		2	4	
**T stage**	2	6	4	1.146	3	1	0.457
	3	26	18		17	14	
	4	61	79		43	48	
**N stage**	1	31	36	1.140	21	24	0.310
	2	40	52		30	33	
	3	22	13		12	6	
**Number of metastatic sites**	0	32	65	0.001	30	35	0.669
	1	48	29		28	24	
	2	7	7		5	4	
	≥3	6	0		0	0	
**TNM stage**	IVa	32	65	0.000	30	35	1.000
	IVb	61	36		33	28	
**Tumor location**	Up	20	29	0.114	11	16	0.271
	Middle	46	57		34	36	
	Down	27	14		16	11	
	Multiple lesions	2	1		2	0	
**HBG (g/L, mean ± SD)**		123.1 ( ± 1.6)	123.3 ( ± 1.8)	0.920	122.1 ( ± 1.9)	124.3 ( ± 2.2)	0.450
**PLT (109/L, mean ± SD)**		318.9 ( ± 11.1)	302.8 ( ± 8.3)	0.248	300.2 ( ± 12.4)	318.8 ( ± 12.2)	0.288
**NEU (109/L, mean ± SD)**		5.9 ( ± 0.3)	5.1 ( ± 0.2)	0.022	5.4 ( ± 0.3)	5.2 ( ± 0.3)	0.691
**LYMPH (10^9^/L, mean ± SD)**		1.9 ( ± 0.1)	2.0 ( ± 0.2)	0.573	2.0 ( ± 0.2)	1.8 ( ± 0.1)	0.338
**ALB (g/L, mean ± SD)**		35.5 ( ± 0.5)	36.7 ( ± 0.4)	0.047	36.8 ( ± 0.6)	37.7 ( ± 0.6)	0.248
**AST (U/L, mean ± SD)**		17.1 ( ± 1.1)	17.2 ( ± 1.0)	0.973	18.1 ( ± 1.7)	17.2 ( ± 1.4)	0.670
**ALT (U/L, mean ± SD)**		25.2 ( ± 1.6)	22.7 ( ± 0.8)	0.159	26.4 ( ± 2.4)	22.3 ( ± 1.0)	0.118
**Urea (mmol/L, mean ± SD)**		4.8 ( ± 0.2)	4.7 ( ± 0.0)	0.786	4.7 ( ± 0.2)	4.5 ( ± 0.2)	0.517
**Creatinine (μmol/L, mean ± SD)**		78.3 ( ± 2.5)	76.9 ( ± 1.4)	0.630	78.1 ( ± 3.1)	76.1 ( ± 1.8)	0.589
**Chemotherapy cycle**	≤3	62	57	0.144	43	35	0.142
	>3	31	44		20	28	

BMI, body mass index; ECOG score, Eastern Cooperative Oncology Group; WBC, white blood cell counts; HGB, hemoglobin level; PLT, blood platelet count; NEU, absolute neutrophil count; LYMPH, absolute lymphocyte count; ALB, albumin level; ALT, alanine aminotransferase level; AST, aspartate aminotransferase level.

### Overall Survival and Progression-Free Survival

**Figure 1:**
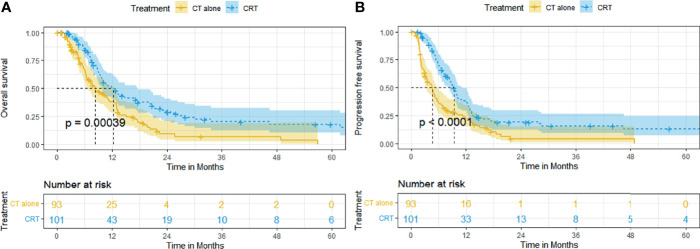
Kaplan–Meier curves of survival in patients with advanced ESCC treated with CRT and CT before PSM: **(A)** overall survival before PSM; **(B)** progression-free survival before PSM.

The median follow-up time was 32.2 months. At the end date of follow-up, 136 (70.1%) patients died and 58 (29.9%) patients were right-censored. Before matching, the median OS and rates of OS at 6, 12, 24, and 60 months were superior in the CRT group to that in the CT group (12.2 months [95% CI, 9.0–15.3], 84.1%, 50.8%, 29.0%, 17.9% vs. 8.2 months [95% CI, 5.4–11.1], 66.0%, 38.3%, 9.1%, 0%, *p* = 0.00039, **Figure 1A**). The median PFS and rates of PFS at 6, 12, 24, and 60 months were also superior in the CRT group to that in the CT group (9.4 months [95% CI, 8.0–10.8], 69.5%, 38.1%, 19.2%, 13.1% vs. 4.7 months [95% CI, 3.5–5.9], 36.6%, 22.4%, 4.1%, 0%, *p* < 0.0001, **Figure 1B**).

**Figure 2:**
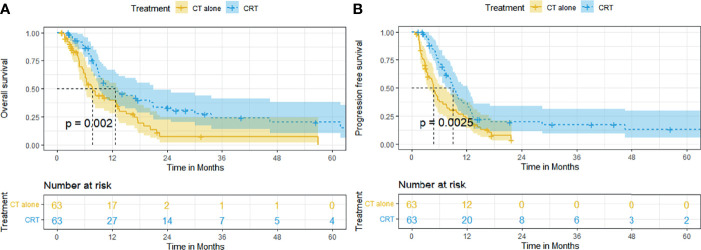
Kaplan–Meier curves of survival in patients with advanced ESCC treated with CRT and CT after PSM: **(A)** overall survival after PSM; **(B)** progression-free survival after PSM.

Adjusting for all baseline factors also demonstrated significant differences between the CRT group and CT group in PFS and OS. The median OS and rates of OS at 6, 12, 24, and 60 months remained superior in the CRT group to that in the CT group (12.5 months [95% CI, 7.1–18.0], 85.9%, 47.4%, 23.4%, 17.3% vs. 7.6 months [95% CI, 5.4–9.8], 63.6%, 39.4%, 7.3%, 0%, *p* = 0.002, **Figure 2A**). The median PFS and rates of PFS at 6, 12, 24, and 60 months also remained superior in the CRT group to that in the CT group (9.0 months [95% CI, 7.6–10.5], 70.9%, 36.5%, 19.7%, 12.9% vs. 4.8 months [95% CI,4.0–5.6], 39.1%, 22.7%, 3.8%, 3.8%, *p* = 0.0025, **Figure 2B**).

### Subgroup Analysis of LocallyAdvanced Disease

**Figure 3:**
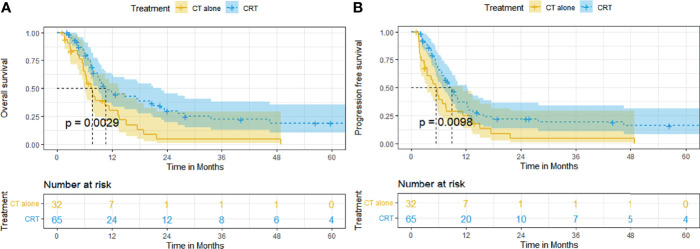
Kaplan–Meier curves of survival in patients with locally advanced ESCC treated with CRT and CT: **(A)** overall survival; **(B)** progression-free survival.

In patients with locally advanced ESCC, the survival outcome of the CRT group was significantly better than that of the CT group. The median OS and rates of OS at 6, 12, 24, and 60 months were superior in the CRT group to that in the CT group (10.5 months [95% CI, 7.3–13.7], 79.8%, 46.6%, 29.8%, 18.9% vs. 7.6 months [95% CI, 5.9–9.3], 65.4%, 30.3%, 4.3%, 0%, *p* = 0.0029, **Figure 3A**). The median PFS and rates of PFS at 6, 12, 24, and 60 months were also superior in the CRT group to that in the CT group (8.9 months [95% CI, 6.9–10.9], 65.7%, 37.3%, 22.0%, 16.0% vs. 5.4 months [95% CI, 3.8–7.0], 42.8%, 25.0%, 4.5, 0%, *p* = 0.0098, **Figure 3B**).

### Subgroup Analysis of Metastatic Disease

**Figure 4:**
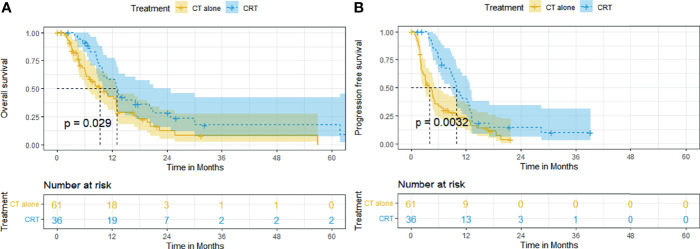
Kaplan–Meier curves of survival in patients with metastatic ESCC treated with CRT and CT: **(A)** overall survival; **(B)** progression-free survival.

CRT also conferred survival benefit to ESCC patients with distant metastasis. The median OS and rates of OS at 6, 12, 24, and 60 months were superior in the CRT group to that in the CT group (12.9 months [95% CI, 10.2–15.7], 91.4%, 58.0%, 28.1%, 17.6% vs. 9.3 months [95% CI, 5.7–13.0], 66.6%, 42.8%, 12.2%, 8.2%, *p* = 0.029, **Figure 4A**). The median PFS and rates of PFS at 6, 12, 24, and 60 months were also superior in the CRT group to that in the CT group (9.9 months [95% CI, 7.9–11.9], 76.5%, 39.9%, 14.7%, 9.8% vs. 4.0 months [95% CI, 2.4–5.7], 33.4%, 20.8%, 3.7%, 3.7%, *p* = 0.0032, **Figure 4B**).

### Survival Analyses on Patients With Locally Advanced and Metastatic Disease

In the entire cohort, both OS (p = 0.75, **Supplementary Figure S1A**) and PFS (p = 0.1, **Supplementary Figure S1B**) did not differ significantly between patients with locally advanced disease and metastatic disease. Similarly, subgroup analysis based on treatment also showed no significant difference in OS (CRT subgroup: p = 0.97, **Supplementary Figure S2A**, CT-alone subgroup: p = 0.28, **Supplementary Figure S2B**) and PFS (CRT subgroup: p = 0.97, **Supplementary Figure S3A**, CT-alone subgroup: p = 0.5, **Supplementary Figure S3B**) between patients with locally advanced disease and metastatic disease.

### Univariate and Multivariate Analysis for Prognostic Factors

Univariable and multivariable Cox analyses for OS and PFS of the entire cohort before PSM are shown in **Supplementary Table S1**. On multivariable analysis of the whole cohort before matching, additional RT, albumin levels, absolute neutrophil count, and chemotherapy cycle were independent prognostic factors for both OS (*p* = 0.041, *p* = 0.000, *p* = 0.001, and *p* = 0.002, respectively) and PFS (*p* = 0.007, *p* = 0.02, *p* = 0.02, and *p* = 0.000, respectively). At the same time, the N stage and number of metastatic independently predicted only PFS (*p* = 0.015 and *p* = 0.007, respectively).

### Treatment Toxicities

Early toxicities that occurred in CRT and CT-alone cohorts were assessed according to the National Cancer Institute Common Toxicity Criteria for Adverse Events, version 4.0 (CTCAE 4.0) (17). The most common adverse events (grades 1–2) of the entire cohort were dysphagia, fatigue, anorexia, nausea, vomit, and hematological toxicities.

Fourteen (22.2%) patients had grade ≥3 radiation esophagitis in the matched CRT group. One (1.6%) patient developed grade 3 radiation pneumonitis 4 months after RT. One (1.6%) patient developed grade 5 upper GI bleeding, and one (1.6%) patient developed grade 3 upper esophageal fistula, both at 3 months after RT. No patient experienced grade ≥3 radiation dermatitis. Twenty-three (36.5%) patients had grade ≥3 leukopenia, 21 (33.3%) had grade ≥3 neutropenia, 8 (12.7%) had grade ≥3 anemia, and 4 (6.4%) had grade ≥3 thrombocytopenia.

In the matched CT-alone group, esophageal fistula occurred in 1 (1.6%) patient after one cycle of CT. Two patients (3.2%) developed grade 3 and 5 upper gastrointestinal bleeding after 2 and 3 cycles of CT, respectively. Four (6.3%) patients had grade ≥3 leukopenia, 6 (9.5%) had grade ≥3 neutropenia, 6 (9.5%) had grade ≥3 anemia, and no patient had grade ≥3 thrombocytopenia.

Patients receiving CRT had a significantly higher incidence of grade ≥3 leukopenia (*p* = 0.000), neutropenia (*p* = 0.000), and thrombocytopenia (*p* = 0.006).

## Discussion

The current study showed that combined definitive dose RT (≥50.4) to the primary tumor with chemotherapy resulted in better OS and PFS than chemotherapy alone in advanced ESCC, even in the presence of metastatic disease, with manageable toxicities. In terms of metastatic EC, extended survival after definitive CRT has been reported by several previous studies. A prospective randomized phase 2 study demonstrated that the CRT was associated with significantly improved median PFS (9.3 vs. 4.7 months, *p* = 0.021) and median OS (18.3 vs. 10.2 months, *p* = 0.001) than CT alone (18). Moreno et al. (19) also suggested that additional RT could derive better survival compared to CT alone with extended 2- and 5-year OS of 6.4% and 2.7%, respectively (p <.001). In a large cohort of 12,683 patients with metastatic EC, Guttmann et al. (13) reported that definitive-dose (≥50.4 Gy) CRT was associated with superior survival compared to CT alone (median OS 8.3 vs. 11.3 months). As shown in **Table 2**, the clinical survival outcomes of the metastatic population in this study were highly consistent with those of previous studies (13, 18–21).

**Table 2:** Definitive radiotherapy combined with chemotherapy versus chemotherapy alone for metastatic esophageal cancer.

Authors	Study design	Number of cases		Chemotherapy cycles	RT prescription, Gy	OS		Median PFS (m)	Median OS (m)
						1 y (%)	2 y (%)		
**Li et al.** (18)	Prospective	CT	30	Mean 3.6	NA	46.6	26.7	4.7	10.2
		CRT	30	Mean 3.8	Median 54.7 (range:50–61)	73.3	43.3	9.3	18.3
**Guttmann et al.** (13)	Retrospective	CT	7229	NA	NA	34	12	NA	8.4
		CRT	2409	NA	>50.4	47	19	NA	11.2
**Moreno et al.** (19)	Retrospective	CT	NA	NA	NA	NA	12.4	NA	NA
		CRT	NA	NA	40–60	NA	18.8	NA	NA
**Lyu et al.** (20)	Retrospective	CT	86	31.4%>2	NA	43	14	6	11
		CRT	55	36.4%>2	Median 56.4 (range: 50–66)	58	25.5	8	14
**Xu et al.** (21)	Retrospective	Non-RT	327	NA	NA	NA	NA	NA	6
		RT	327	NA	NA	NA	NA	NA	10
**Present study**	Retrospective	CT	61	Median 3	NA	42.8	12.2	4.0	9.3
		CRT	36	Median 3	Median 60 (range: 50–66)	58.0	28.1	9.9	12.9

NA, not applicable.

In the current study, most (93.8%) patients with metastatic disease had only one or two metastatic sites. We found no statistical difference in survival results between the locally advanced disease and metastatic disease. These results highlight that for advanced ESCC patients with low systemic tumor load, survival is most threatened by the failure of local control of the primary tumor. On the one hand, additional RT for primary tumor can effectively shrink the primary tumor and reduce dysphagia resulting from esophageal stricture. By increasing oral nutritional intake, RT may improve response rates, performance status, and long-term survival (22, 23). In the current study, multivariate analysis revealed that albumin level (*p* = 0.000) before treatment was an independent prognostic factor for OS, further illustrating the importance of the nutritional state for patients with advanced EC. On the other hand, aggressive RT for primary tumor can reduce life-threatening events, including airway stenosis either by external compression or by direct tumor growth into the airways, fistula, perforation, and massive bleeding. It is reported that external beam RT could provide significantly more effective relief of pain and tumor-related complications for metastatic EC compared to esophageal stent placement (5).

Based on modern radiotherapy techniques, definitive RT (≥50.4) to the primary tumor may confer more significant survival benefits than palliative RT (≤50.4 Gy) in patients with advanced EC. In the palliative setting, low-dose radiotherapy of less than 50.4 Gy is commonly used to relieve symptoms such as dysphagia, pain, and bleeding (5). However, the toxicity resulting from low-dose radiotherapy with chemotherapy may overweight the clinical benefit it confers. In a phase 3 randomized controlled trial, concurrent palliative RT (20 Gy in 5 fractions or 30 Gy in 10 fractions) did not derive additional benefit on survival for advanced EC patients with self-expanding metal stent placement (median OS: 19.7 weeks with usual care vs. 18.9 weeks with EBRT, *p* = 0.07) (24). Another multicenter randomized controlled trial (TROG 03.01) also indicated that palliative CRT (30–35 Gy in 10–15 fractions) failed to significantly relieve dysphagia and prolong survival (median OS: 6.9 vs. 6.7 months, *p* = 0.88) compared to RT alone, with increased toxicity (grade 3–4 acute toxicity: 36% vs. 16%, *p* = 0.0017) (25). Guttmann et al. (13) reported that compared to CT alone, definitive-dose (≥50.4 Gy) CRT was associated with superior survival (median OS 11.2 vs. 8.4 months, *p* ≤ 0.001), while palliative dose (<50.4 Gy) CRT was associated with slightly inferior outcomes (median OS: 7.6 vs. 8.4 months, *p* = 0.004).

In the current study, patients in the definitive CRT group received a high radiation dose of 56–66 Gy, with most patients receiving radiation dose ≥60 Gy (97 in 101, 96%). The precise dose of definitive RT remains controversial. The landmark RTOG94-05 trial (26) failed to demonstrate the superiority of high-dose (64.8 Gy) over conventional-dose (50.4) concurrent CRT, providing a theoretical basis for the standard RT paradigm for EC in Europe and America (10). According to this study, high-dose RT was not able to increase survival time (median OS: 13.0 vs. 18.1 months, *p* > 0.05) and regional control (56% vs. 52%, *p* > 0.05), but rather it seemed to increase the toxicity and higher treatment-related mortality rate. Notably, patients with squamous cell carcinoma account for the vast majority of the participants in this trial. Differently, radiation doses above 60 Gy are more frequently adopted in Asia. Several studies proposed that high-dose concurrent CRT of ≥60Gy could improve clinical outcomes compared with standard dose (50–54 Gy) in EC (27, 28), especially ESCC. A pooled analysis reported that in cisplatin-based definitive concurrent CRT, high-dose RT (≥60 Gy) was associated with significantly higher local regional recurrent rates (22% vs. 30%, p = 0.01) and distant failure rates (13% vs. 25%, *p* < 0.000) compared with conventional-dose RT (50–54 Gy) in ESCC patients (27). However, according to the ARTDECO study, an increase in RT dose to 61.6 Gy did not result in better local control over 50.4 Gy for both adenocarcinoma and squamous cell carcinoma (29). The optimal radiation dose of definitive CRT for EC merits further investigation, especially in a metastatic setting.

Safety findings in the current study were consistent with the known safety profile of CRT and CT alone (30–32). Significantly higher incidences of grade ≥3 hematological toxicities were observed in patients treated with CRT. What is more, additional definitive RT has led to severe radiation-related toxicities such as radiation esophagitis in certain patients, but with an acceptable incidence rate (14/63, 22.3%). Advancement of modern RT techniques, such as intensity-modulated RT (IMRT), volume modulated arc therapy (VMAT), and image-guided RT (IGRT), has improved the safety of definitive RT with precise radiation delivery while reducing the dose to organ at risk. Therefore, standard CRT may be a better option in well-selected metastatic ESCC patients who are in good general condition and had low burden of distant metastases. It should be considered after patients are fully informed of the risk benefits.

In the rapid development of immunotherapeutic strategies, local radiotherapy may play a more significant role in metastatic EC. Anti-programmed death 1 (PD-1)/programmed death ligand-1 (PD-L1) therapies are currently the research hotspot and have demonstrated durable antitumor activity in patients with advanced EC (32–34). According to the recently published ESCORT-1st randomized clinical trial, camrelizumab combined with chemotherapy significantly improved OS (15.3 vs. 12.0 months, *p* = 0.001) and PFS (6.9 vs. 5.6 months, *p* <; 0.001) compared with chemotherapy alone as first-line treatment in patients with advanced ESCC (14). As previously demonstrated in various cancers (such as non-small cell lung cancer (NSCLC) and metastatic melanoma), the combination of radiotherapy and immune checkpoint inhibitors could promote systemic antitumor immunity and abscopal effect (35, 36). This novel approach also represents an effective therapeutic option in advanced EC, and pertinent clinical trials are currently ongoing (37). A phase 3 study (KEYNOTE-975) of definitive CRT plus pembrolizumab in advanced EC is now in the recruiting phase (NCT04210115). The dual primary endpoints are OS and event-free survival, which is highly anticipated (38).

The present study had several limitations. Firstly, propensity score matching was used to reduce selection bias in this study. However, this led to the selection of patients and thereby decreased the sample size. Secondly, due to the retrospective nature of this study, data on quality of life were not available to us. Thirdly, we did not consider the changes in objective factors during the long-term period, such as increased applications of PET-CT, radiotherapy techniques, and chemotherapy regimens.

In conclusion, additional definitive-dose radiotherapy was associated with improved clinical outcomes in locally advanced and metastatic ESCC. Therefore, more consideration should be given to its application in the metastatic setting.

## Data Availability Statement

The original contributions presented in the study are included in the article/**Supplementary Material**. Further inquiries can be directed to the corresponding author.

## Ethics Statement

The studies involving human participants were reviewed and approved by the Institutional Ethics Committee of Guangxi Medical University Cancer Hospital. The ethics committee waived the requirement of written informed consent for participation.

## Author Contributions

Data curation: L-QL, Y-DW, and W-DZ, W-WM. Formal analysis: L-QL and Q-GF. Writing—original draft: L-QL and Q-GF. Writing—review and editing: T-SS. Funding acquisition: T-SS. All authors contributed to the article and approved the submitted version.

## Funding

This research was supported in part by the Guangxi Natural Science Foundation (CN) (2020GXNSFAA297171), China International Medical Foundation-Tumor Precise Radiotherapy Spark Program (2019-N-11-01), Guangxi Medical University Training Program for Distinguished Young Scholars, and Guangxi BaGui Scholars’ Special Fund.

## Conflict of Interest

The authors declare that the research was conducted in the absence of any commercial or financial relationships that could be construed as a potential conflict of interest.

## Publisher’s Note

All claims expressed in this article are solely those of the authors and do not necessarily represent those of their affiliated organizations, or those of the publisher, the editors and the reviewers. Any product that may be evaluated in this article, or claim that may be made by its manufacturer, is not guaranteed or endorsed by the publisher.
