# Supplementary material for: Chemoradiotherapy Versus Chemotherapy Alone for Advanced Esophageal Squamous Cell Carcinoma: The Role of Definitive Radiotherapy for Primary Tumor in the Metastatic Setting
Source: Front Oncol. 2022 Mar 30;12:824206. doi: 10.3389/fonc.2022.824206 (PMC9005791; doi:10.3389/fonc.2022.824206)
Supplement: Supplementary file 1 [file DataSheet_1.docx]

Supplementary Material

1. **Supplementary Table S1. Univariable and multivariable Cox analyses for OS and PFS in the entire cohort.**

|  |  | OS |  | | PFS |  | |
| --- | --- | --- | --- | --- | --- | --- | --- |
| Factor | **level** | Univariable | Multivariable | | Univariable | Multivariable | |
|  |  | P value | P value | HR | P value | P-value | HR |
| Additional RT | no | 0.000 | 0.041 | 1.463 | 0.000 | 0.007 | 1.586 |
|  | yes |  |  |  |  |  |  |
| Gender | male | 0.079 |  |  | 0.021 | 0.052 | 1.914 |
|  | female |  |  |  |  |  |  |
| Age |  | 0.358 |  |  | 0.873 |  |  |
| BMI |  | 0.083 |  |  | 0.225 |  |  |
| ECOG score | 0 | 0.585 |  |  | 0.190 |  |  |
|  | 1 |  |  |  |  |  |  |
|  | 2 |  |  |  |  |  |  |
| Smoking | no | 0.541 |  |  | 0.469 |  |  |
|  | yes |  |  |  |  |  |  |
| Drinking | no | 0.278 |  |  | 0.406 |  |  |
|  | yes |  |  |  |  |  |  |
| Family history | no | 0.039 | 0.09 | 0.49 | 0.093 |  |  |
|  | yes |  |  |  |  |  |  |
| T stage | 2 | 0.888 |  |  | 0.857 |  |  |
|  | 3 |  |  |  |  |  |  |
|  | 4 |  |  |  |  |  |  |
| N stage | 1 | 0.091 |  |  | 0.009 | 0.015 | 0.569 |
|  | 2 |  |  |  |  |  | 0.513 |
|  | 3 |  |  |  |  |  |  |
| Number of metastatic sites | 0 | 0.183 |  |  | 0.000 | 0.007 | 0.215 |
|  | 1 |  |  |  |  |  | 0.289 |
|  | 2 |  |  |  |  |  | 0.227 |
|  | ≥3 |  |  |  |  |  |  |
| TNM stage | IVa | 0.747 |  |  | 0.104 |  |  |
|  | IVb |  |  |  |  |  |  |
| Tumor location | up | 0.291 |  |  | 0.421 |  |  |
|  | middle |  |  |  |  |  |  |
|  | down |  |  |  |  |  |  |
|  | Multiple lesions |  |  |  |  |  |  |
| HBG |  | 0.708 |  |  | 0.636 |  |  |
| PLT |  | 0.062 |  |  | 0.078 |  |  |
| NEU |  | 0.000 | 0.001 | 1.12 | 0.000 | 0.02 | 1.079 |
| **LYMPH** |  | 0.857 |  |  | 0.816 |  |  |
| ALB |  | 0.000 | 0.000 | 0.93 | 0.001 | 0.02 | 0.958 |
| AST |  | 0.801 |  |  | 0.793 |  |  |
| ALT |  | 0.207 |  |  | 0.767 |  |  |
| Urea |  | 0.980 |  |  | 0.668 |  |  |
| Creatinine |  | 0.773 |  |  | 0.761 |  |  |
| Chemotherapy cycle | ≤3 | 0.000 | 0.002 | 1.800 | 0.000 | 0.000 | 1.998 |
|  | >3 |  |  |  |  |  |  |

# Supplementary Figures


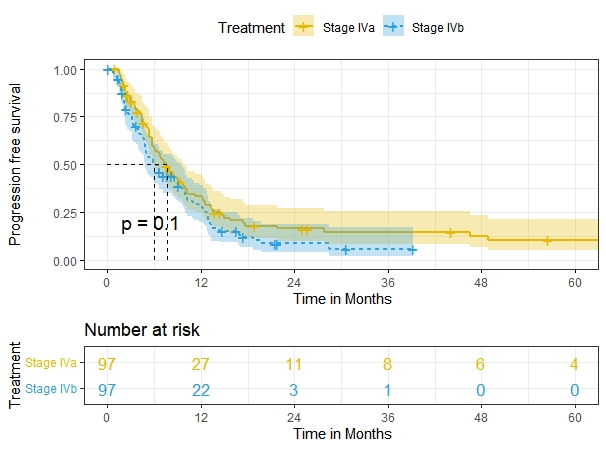

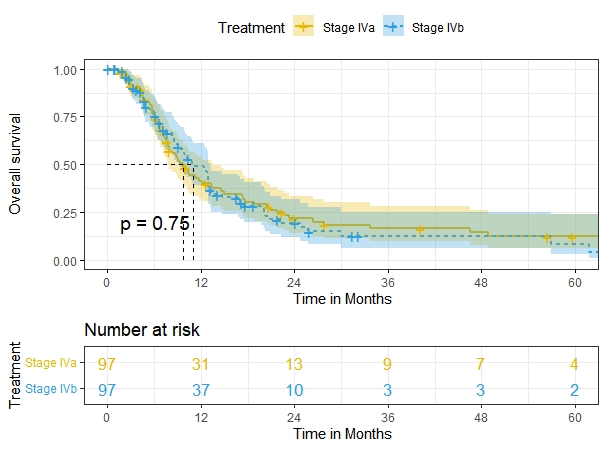
**Figure S1A Figure S1B**

1. **
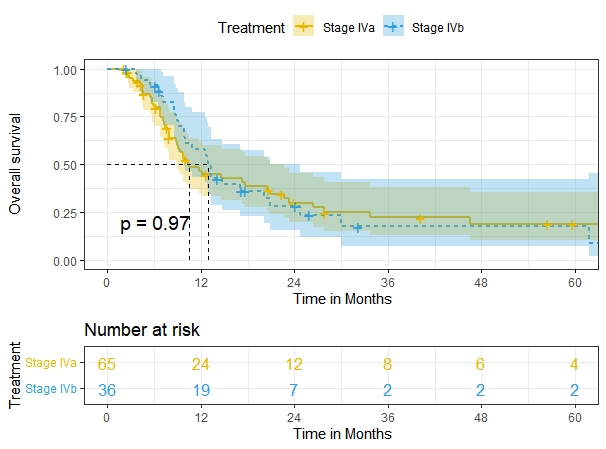
**Kaplan-Meier curves of survival of patients with locally advanced disease (Stage IVa) and metastatic disease (Stage IVb) across the entire cohort: (A) Overall survival; (B) Progression-free survival
2. Kaplan-Meier curves of survival of patients with locally advanced disease (Stage IVa) and metastatic disease (Stage IVb) in the CRT subgroup: (A) Overall survival;


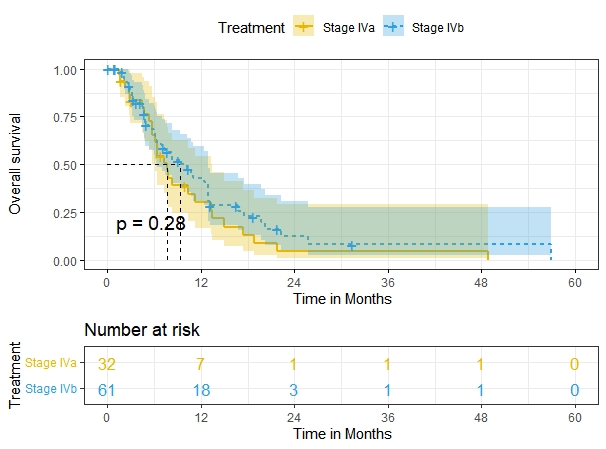
**2.** Kaplan-Meier curves of survival of patients with locally advanced disease (Stage IVa) and metastatic disease (Stage IVb) in the CT alone subgroup: (B) Overall survival;


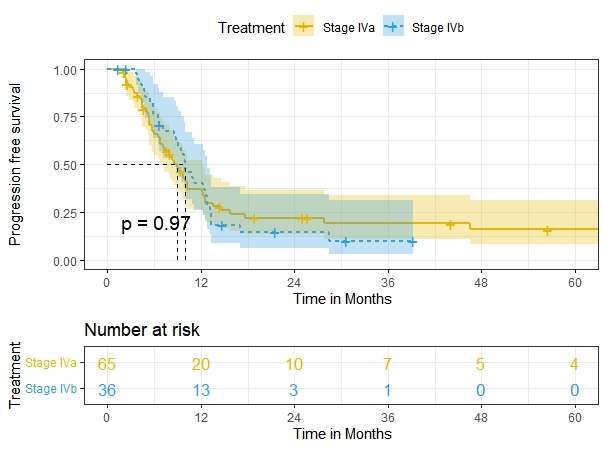


1. Kaplan-Meier curves of survival of patients with locally advanced disease (Stage IVa) and metastatic disease (Stage IVb) in the CRT subgroup: (A) Progression-free survival;


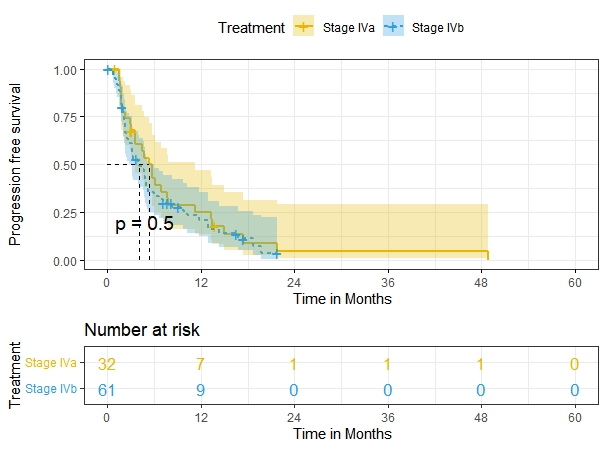


**3.** Kaplan-Meier curves of survival of patients with locally advanced disease (Stage IVa) and metastatic disease (Stage IVb) in the CT alone subgroup: (B) Progression-free survival;
